# Mass molecular testing for COVID19 using NGS-based technology and a highly scalable workflow

**DOI:** 10.1038/s41598-021-86498-3

**Published:** 2021-03-29

**Authors:** Fernanda de Mello Malta, Deyvid Amgarten, Felipe Camilo Val, Murilo Castro Cervato, Bruna Mascaro Cordeiro de Azevedo, Marcela de Souza Basqueira, Camila Oliveira dos Santos Alves, Maria Soares Nobrega, Rodrigo de Souza Reis, Pedro Sebe, Michel Chieregato Gretschischkin, Diego Delgado Colombo de Oliveira, Carolina Naomi Izo Nakamura, Pedro Lui Nigro Chazanas, João Renato Rebello Pinho

**Affiliations:** Laboratorio de Técnicas Especiais, Hospital Israelita Albert Einstein, São Paulo, Brazil

**Keywords:** Next-generation sequencing, Infectious-disease diagnostics, Microbial genetics, Virology, Genomics, RNA sequencing

## Abstract

Since the first reported case of the new coronavirus infection in Wuhan, China, researchers and governments have witnessed an unseen rise in the number of cases. Thanks to the rapid work of Chinese scientists, the pathogen now called SARS-CoV-2 has been identified and its whole genome was deposited in public databases by early January 2020. The availability of the genome has allowed researchers to develop Reverse Transcription—Polymerase Chain Reaction (RT-PCR) assays, which are now the gold-standard for molecular diagnosis of the respiratory syndrome COVID19. Because of the rising number of cases and rapid spreading, the world has been facing a shortage of RT-PCR supplies, especially the ones involved in RNA extraction. This has been a major bottleneck to increase testing capacity in many countries that do not significantly manufacture these supplies, such as Brazil. Additionally, RT-qPCR scalability is highly dependent on equipment that usually performs testing of 96 samples at a time. In this work, we describe a cost-effective molecular NGS-based test for diagnosis of COVID19, which uses a single-step RNA extraction and presents high scalability and accuracy when compared to the gold-standard RT-qPCR. A single run of the NGS-based test using the Illumina NextSeq 550 mid-end sequencing equipment is able to multiplex 1,536 patient’s samples, providing individual semi-qualitative results (detected, not detected). Detected results are provided with fragments per million (FPM) values, which was demonstrated to correlate with RT-qPCR Cycle Threshold (CT) values. Besides, usage of the high-end Illumina Novaseq platform may yield diagnostic for up to 6144 samples in a single run. Performance results when compared with RT-qPCR show general accuracy of 96%, and 98% when only samples with CT values (gene N) lower than 30 are considered. We have also developed an online platform, termed VarsVID, to help test executors to easily scale testing numbers. Sample registering, wet-lab worksheets generation, sample sheet for sequencing and results’ display are all features provided by VarsVID. Altogether, these results will contribute to control COVID19 pandemics.

## Introduction

In December 2019, it was reported in Wuhan City, Hubei province in China, cases of pneumonia of unknown etiology. In 7th January 2020, the causative agent was identified from throat swab samples in a study conducted by the Chinese Center for Disease Control and Prevention (CCDC)^1^. The new coronavirus was provisionally named 2019 novel coronavirus (2019-nCoV)^2,3^. Coronaviruses, from the family *Coronaviridae*, are enveloped single-strand RNA viruses with genomes ranging from 26 to 32 kilobases in size. Most members of the coronaviruses family have similar genome organization and expression, usually composed by ten or more nonstructural proteins (nsp proteins) and by the structural proteins spike (S), envelope (E), membrane (M), and nucleocapsid (N) (3). Through Next-Generation Sequencing technology (NGS), the virus complete genome was described and phylogenetically classified as belonging to the genus *Betacoronavirus*. Thereafter, 2019-nCOV was named SARS-CoV-2^1,4^.

In March 2020, the WHO (World Health Organization) declared COVID-19 as pandemic^5^, and have issued guidelines about clinical and epidemiological findings, stating that extensive laboratory tests should be performed^6^. Since then, there has been a robust scientific response and a global search for the best diagnostic tool to identify patients infected by SARS-CoV-2. Several laboratory methods have been used to detect the etiological agent of this respiratory syndrome, as for instance enzyme-linked immunosorbent assay and nucleic acid hybridization. Moreover, early publication of SARS-COV-2 genome has allowed development of molecular tests based on reverse transcription polymerase chain reaction, widely known as RT-qPCR. Months into the pandemics now, RT-qPCR has been the gold standard technique for molecular identification of the virus and for diagnosis of COVID19.

NGS technology has revolutionized the genomics field and has been providing a novel and effective way to screen samples and detect pathogens. In association with bioinformatics tools, this technology is changing the way as research and diagnostic centers can respond to infectious disease outbreaks. This progress is paving the way for new approaches and for improving our knowledge about the disease origin, occurrence and transmission, through a large-scale genomic approach. Over 16,000 SARS-CoV-2 genome sequences have been deposited in public repositories until May 2020, and genome sequencing is helping researchers to understand SARS-COV-2 haplotypes distribution, mutations and phylogeny^7–9^.

In a pandemic scenario, it is essential to carry out population mass testing to prevent infections rapid spread and minimize mortality rates. In response to this need, we have developed an NGS-based diagnostic test for the new coronavirus, which presents 96% of accuracy in paired analyses with the RT-qPCR gold standard. Our NGS-based test is highly scalable, having the ability to perform up to 1536 samples simultaneously on a mid-end NextSeq 550 Illumina sequencing equipment. This number is 16 times more than what is done today by the gold standard RT-qPCR technique and it may be even more expanded considering the use of a high-end Novaseq 6000 Illumina equipment (3072–6144 samples in a single run). This new technology expands the global diagnostic capacity, enabling rapid initiation of treatment and isolation of the infected, altogether contributing to control COVID19 pandemics.

## Results

### General workflow

**Figure 1:**
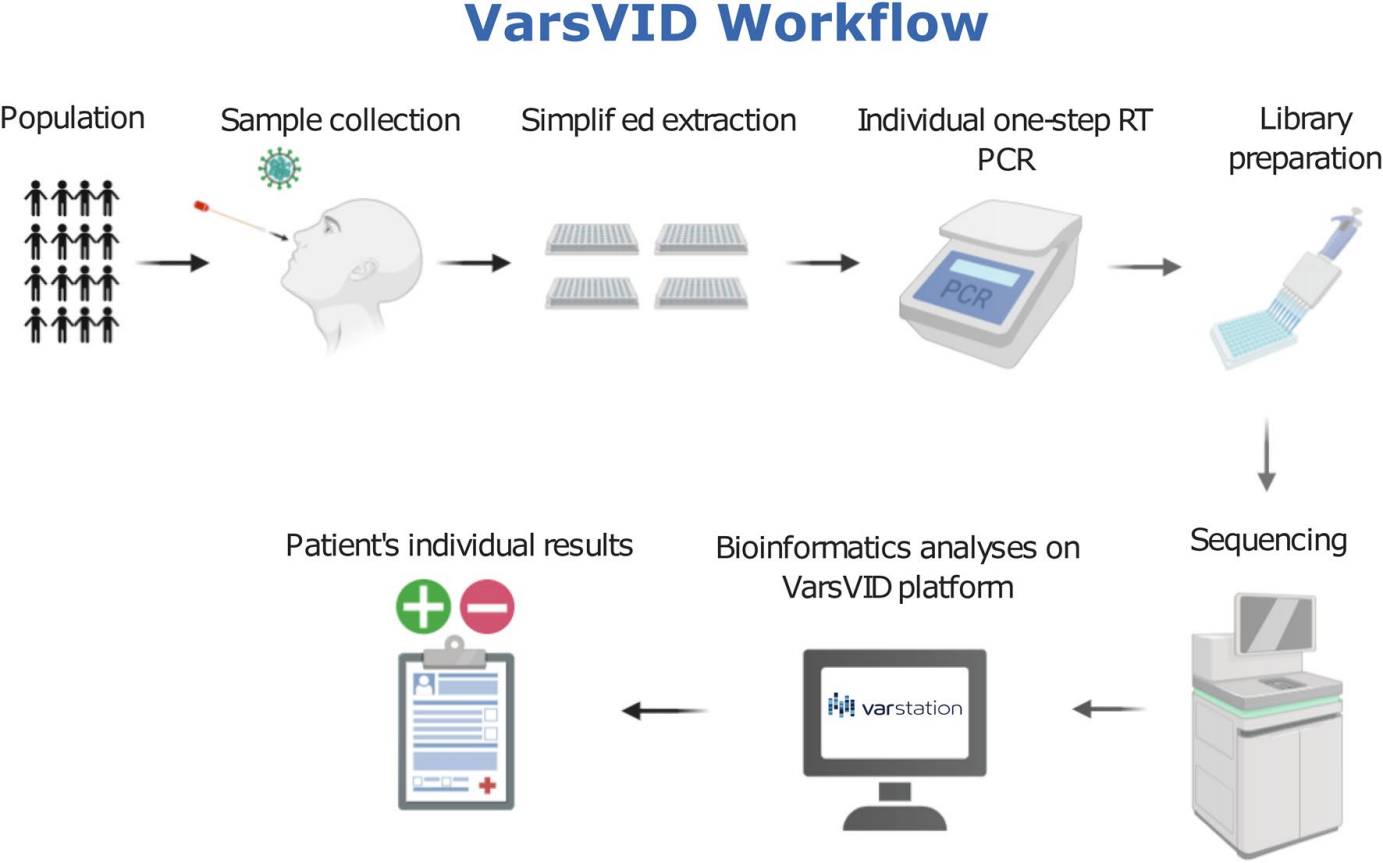
Panoramic view of the COVID19 NGS-based test reported in this work. Created with BioRender.com.

We have developed a complete workflow to execute the NGS-based testing, including wet-lab and bioinformatics analyses, as shown in Fig. 1. This workflow is highly scalable in order to avoid bottlenecks and to increase testing capacity. The wet-lab steps may be carried out by pipetting robots to minimize errors and to reduce hands-on. From DNA to results, the total hands-on time varies depending on the input number of samples and sequencing platform used (see Supplementary Table S1 for detailed information of turn-around time). For this reason, the number of technicians and equipment must be adjusted depends on sample size to keep the total process time in a median of 48 h, running 1.500, 3.000 or 6000 samples/run (from 22 to 31 h just for sequencing using Novaseq 6000 and NextSeq 550 Illumina equipment, respectively). Bioinformatics analyses may be performed by a time and cost-efficient pipeline implemented on Varstation online platform (https://varstation.com/) using Amazon Web Services (AWS).

### Performance and limit of detection

In this work, we show a new amplicon-based methodology to amplify highly conserved regions of the SARS-CoV-2 genome and to uniquely identify these fragments using NGS sequencing. Two types of RNA extraction were used to test our NGS protocol: (1) Simple one-step RNA extraction with QuickExtract (Lucigen, WI, USA) and a regular kit extraction using MagMAX Viral/Pathogen II Nucleic Acid Isolation Kit (MVP II; Thermo Fisher).

**Figure 2:**
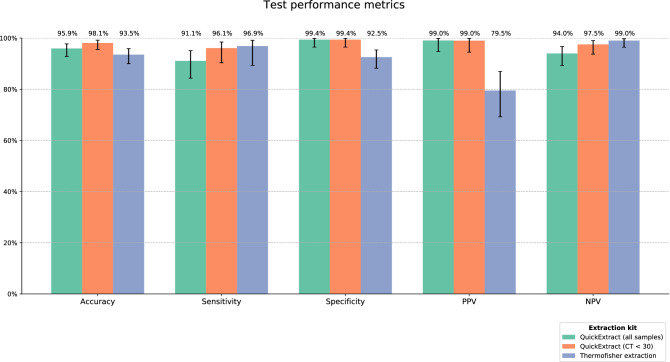
Performance results for the NGS approach in three different scenarios: (1) One-step RNA extraction using QuickExtract for all samples; (2) One-step RNA extraction using QuickExtract for samples with CT values lower than 30; (3) Regular ThermoFisher kit RNA extraction using MagMAX Viral/Pathogen II Nucleic Acid Isolation Kit. Error bars indicate 95% confidence intervals for the binomial proportions, calculated using Wilson score interval.

A total of 269 clinical samples were extracted with QuickExtract and tested using the NGS approach. Performance results are summarized as follows. From 112 samples which were positive in RT-qPCR, 102 were confirmed by NGS. From 156 samples RT-qPCR that tested negative, NGS results confirmed 155. These numbers show that the NGS-based technique has overall accuracy of 95.9%, with 91.1% and 99.4% of sensitivity and specificity, respectively. Based on these results we also calculated the positive predictive value (PPV) and the negative predictive value (NPV). PPV value was 99% (102 NGS true positive and 1 NGS false positive) and NPV value was 94% (156 true negatives and 10 false negatives). We noted that most false negative results presented RT-qPCR Cycle Threshold (CT) values higher than 30 (6 false negatives). Sensitivity and specificity for samples with CT values lower than 30 were 96.1% and 99.4%, respectively. Accuracy for samples with CT values lower than 30 hitched 98.1% (Fig. 2).

A total of 278 samples were extracted with MagMAX Viral/Pathogen II Nucleic Acid Isolation Kit (MVP II; Thermo Fisher) and tested using our NGS protocol, resulting in 93.5% of overall concordance with RT-qPCR. As we will explore in the Discussion section, a decrease in specificity was observed using regular nucleic acids extraction (16 false positives). We believe these results are probably due to a higher limit of detection of the NGS technique when compared with RT-qPCR. From 64 negative samples in NGS, only 2 yielded a positive result in RT-qPCR. Performance results are summarized in Fig. 2 and detailed contingency tables are shown in Table 1.

**Table 1:** Contingency table showing results in absolute count for each extraction method.

RT-qPCR/NGS	Quick extract extraction			Thermo fisher extraction		
	Positive	Negative	Total	Positive	Negative	Total
Positive	102	10	112	198	16	224
Negative	1	156	157	2	62	64
Total	103	166	269	200	78	278

Rows are actual RT-PCR results and columns are NGS results.

NGS testing of the AMPLIRUN Coronavirus RNA quantified commercial SARS-COV-2 control (Vircell Microbiologists, Spain), shows that dilutions from 1 × 10^3^ copies/mL to 1 × 10^5^ copies/mL were successfully identified by the technique presented in this work. To further assess Limit of Detection (LoD) of the test, we have tested 71 samples at dilutions 1 × 10^3^ copies/mL originated from a high titer SARS-CoV-2 culture in Vero E6 cells. 68 samples (95.7%) were successfully identified by the NGS-based technique. Therefore, we defined 1 × 10^3^ copies/mL (or 1 copy/µL) as the limit of detection of the test.

### NGS coverage

**Figure 3:**
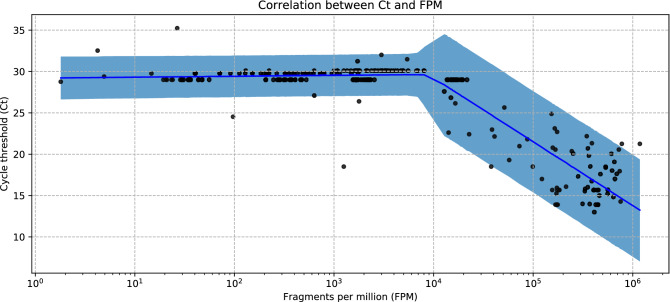
Correlation between RT-PCR cycle threshold and Fragments per Million. The blue line indicates the posterior median of the Ct for each value of FPM. Blue shade indicates 95% posterior predictive interval (that is, 95% of new samples are expected to fall within this range).

NGS coverage was assessed for all positive samples, and average SARS-CoV-2 target NGS coverage per sample was 25,172x. Additionally, we calculated a measure of fragments per million (FPM), which is suitable to compare coverage among different samples and sequencing runs. Positive samples presented average FPM of 2.14 × 10^5^, while average CT for gene N was 24.4. In order to evaluate the relationship between CT and FPM values, a piecewise linear regression model was fit (Fig. 3). FPM values were log-transformed, since it is in principle a linear measure of viral load, while CT is a logarithmic measure. The analysis was carried out on PyMC3, an open-source Bayesian inference framework. Further details about the statistical model and MCMC sampling are available as Supplemental Table S2. The correlation between the observed CT and the ones estimated by the model is 0.926.

FPM values of the quantified spike-in controls presented low variance among samples tested, averaging 1.42 × 10^8^ with standard deviation of ± 0.58 × 10^8^. Therefore, we propose this new metric to be associated with qualitative NGS results in the same way as CT values are associated with RT-qPCR. FPM values may provide patients and physicians an important semi-quantitative idea of viral loads to guide clinical responses. It is important to note, though, that FPM values may vary according to the viral target (Spike, N, Matrix, etc.) and that one target may be more sensitive than others as occurs with RT-qPCR probes.

### Automation and user-friendly results using cloud resources

**Figure 4:**
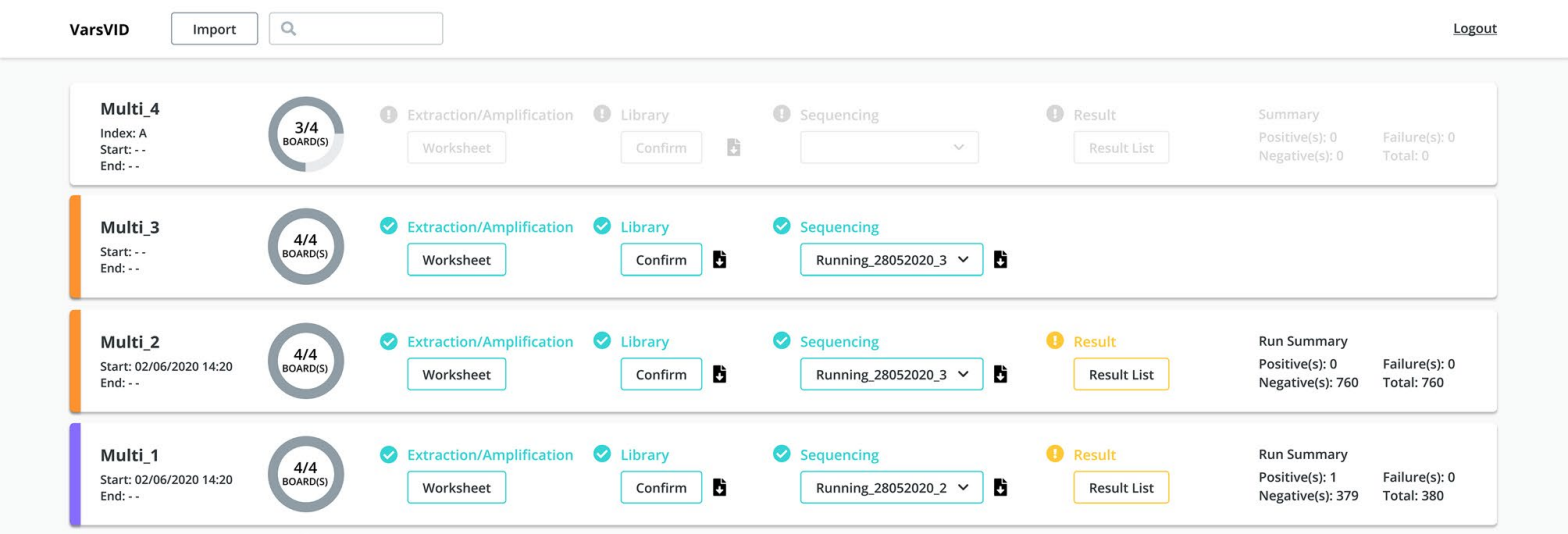
Front-end of the VarsVID online platform, where users can register samples, check wet-lab steps completion, and inspect results. Print screen obtained directly from the platform.

As this test was intended to process thousands of samples per run, we anticipated an enormous struggle to organize and perform wet-lab assays. To address this potential bottleneck, we have developed an online platform called VarsVID to automate the entire process of sample registrations and generation of worksheets. VarsVID is a Varstation feature (https://varstation.com/). Wet-lab professionals will only need to provide a CSV file with sample’s relevant information in order to plan a run and generate worksheets. These worksheets were intended to guide the wet-lab professional and to avoid planning mistakes (see Supplementary Table S6). VarsVID will also provide a SampleSheet for Illumina sequencing, so that the professional will not waste unnecessary time registering hundreds of libraries in the Illumina basespace platform. Bioinformatics analysis was also automated on the VarsVID platform, which was planned to be cloud-based and highly elastic according to a user’s needs. These efforts resulted in the analyses of thousands of samples FASTQs in a matter of minutes. Finally, VarsVID provides user-friendly online results, as well as the possibility of generating csv files for further processing (Fig. 4).

## Discussion

While there is a global struggle to control the SARS-CoV-2 pandemic, it is fundamental that new diagnostic methodologies allow population testing. Our test was developed considering the best way to guarantee access to accurate molecular diagnosis, without compelling bottlenecks of supplies such as RNA extraction kits. This proposed methodology uses simple and commonly available reagents, providing an accurate and cost-effective virus detection solution. Along with the VarsVID platform, this newly developed NGS-based test is able to detect SARS-CoV-2 in higher scale and lower costs when compared with RT-qPCR, which is considered the gold standard methodology for molecular diagnosis of COVID19. To provide an example, multiplexing 1536 samples can cost as little as 15 USD/per sample for the entire procedure. It is important to note that our approach is aligned with other alternative solutions for COVID19 diagnosis, such as RT-lamp^10^, mass spectrometry^11^ and CRISPR based diagnostic^12^.

We demonstrate that this proposed workflow is highly scalable for three mains reasons. First, labor intense steps were automated using pipet robots and an online platform that perform sample registering and generates sample sheets for sequencing. Second, work-around times for each step were estimated in different scenarios, so that enough lab technicians would be available for non-automatable steps. Last, supplies used in the protocol were selected to avoid bottlenecks and overlap with RT-qPCR test reagents. Global demand of one-step extraction kits, Taq-polymerases and sequencing reagents were not significantly affected during the pandemics.

We show high concordance with RT-qPCR, even when a simple one-step procedure was used for RNA extraction. When a regular kit approach was used, 16 non-agreements were observed as false positives. However, we acknowledge the possibility of the NGS-based technique combined with regular kit extraction being more sensitive to detect SARS-CoV-2 RNA than RT-qPCR. We tested 2 of the 16 alleged false positives using a more sensitive RT-qPCR technique (Xpert Xpress SARS-CoV-2, Cepheid) and both results came positive with CT values above 40. Therefore, we conjecture that the NGS technique presented in this work has a limit of detection of fewer copies/reaction than gold standard RT-qPCR.

By showing that FPM values correlate with RT-qPCR CT values, we provide an alternative to outputting solely qualitative NGS results. These FPM values may be released along with qualitative results, as relative viral load. A function was deduced from empirical data to approximate CT values, given FPMs. Nevertheless, we emphasize that CT values are not suitable for an NGS-based test and we argue that FPM should be used instead. This is a measure whose variations have been extensively used in NGS experiments, such as transcriptome and single cell sequencing^13,14^.

## Methods

### Samples and previous results

A total of 336 samples with previous results for COVID19 RT-qPCR were de-identified and selected from routine testing at the Laboratorio de Tecnicas Especiais, Hospital Israelita Albert Einstein (São Paulo, Brazil). The samples used were received and processed in a period shorter than 24 h, during this period they were kept under refrigeration. RT-qPCR assay performed was XGEN MASTER COVID-19 kit (Mobius Life Science), which uses a protocol for amplification of fragments of the genes N and ORF1ab, following the manufacture instruction.

From the 336 samples, 160 tested positive for SARS-COV-2 with Cts values for gene N ranging from 13 to 35.24. Remaining 176 samples tested negative. We have also included negative plaque-controls to assess cross-contamination among plaques or other sequencing artefacts. Finally, AMPLIRUN Coronavirus RNA quantified commercial SARS-COV-2 control (Vircell Microbiologists, Spain) was serially diluted and tested for assessing limit of detection (LoD). Further testing of the LoD was performed in diluted samples from a high titer SARS-CoV-2 culture isolated and quantified as described^15^. Clinical samples presented in this work were collected directly from patients for diagnosis. The research project was submitted for ethical evaluation to the Institutional Review Board (IRB) of Hospital Israelita Albert Einstein and was approved on July 23, 2020 (report #4.178.076). IRB also approved our request for exemption from application of the informed consent form. All methods and protocols were carried out in accordance with relevant guidelines and regulations.

### RNA extractions and internal controls

The total set of 336 samples was further divided in two subsets of samples to test or NGS approach using two different nucleic acids extraction methodologies. The first approach used was QuickExtract DNA Extraction Solution (n = 269 samples; Lucigen, WI, USA) according to the manufacturer's instructions. This method requires only heat treatment for 8 min direct to a plaque where samples are placed. The second, MagMAX Viral/Pathogen II Nucleic Acid Isolation Kit (n = 278 samples; Thermo Fisher). It is a semi-automated procedure, taking about 90 min to extract 96 samples. 201 of the 336 samples were placed in both subsets. MS2 phage RNA spike-in was added to function as an additional internal control of true negative samples, i.e., negative samples that do not yield MS2 sequences after bioinformatics analyses will be automatically placed for repetition.

### Amplicon generation by RT-PCR

RT-PCR reactions were performed individually using SuperScript™ III One-Step RT-PCR (Invitrogen) with a combination of multiplex primers designed to amplify highly conserved regions of SARS-CoV-2 and MS2 control genomes. Several target and control primers were tested for efficiency and multiplex compatibility (See Supplementary Tables S3, S4 and S5 for primer sequences and combinations used). A total of four different combinations were used (target A + target B + control C), which will be pooled afterwards in a single library well. Assays were carried out following standard protocols recommended for the kit: 11 µL template RNA, 2 µL SuperScript™ III RT/Platinum™ Taq Mix (Thermo Fisher Scientific, Waltham, MA), 25 µL 2X Reaction Mix (Thermo Fisher Scientific, Waltham, MA), 1 µL of each primer 10 µM (multiplex primer 1 forward, multiplex primer 1 reverse, multiplex primer 2 forward, multiplex primer 2 reverse), 0.5 µL of internal control primer forward 10 µM, 0.5 µL of internal control primer reverse 10 µM and 7 µL nuclease-free water for a final reaction volume of 50 µL. The RT-PCR reactions were incubated using the following cycling conditions: 56 °C for 15 min, followed by one cycle of 94 °C for 2 min, followed by 40 cycles of 94 °C for 15 s, 65 °C for 30 s and 72 °C for 5 s and followed by one cycle of 72 °C for 5 min.

### Library generation

**Figure 5:**
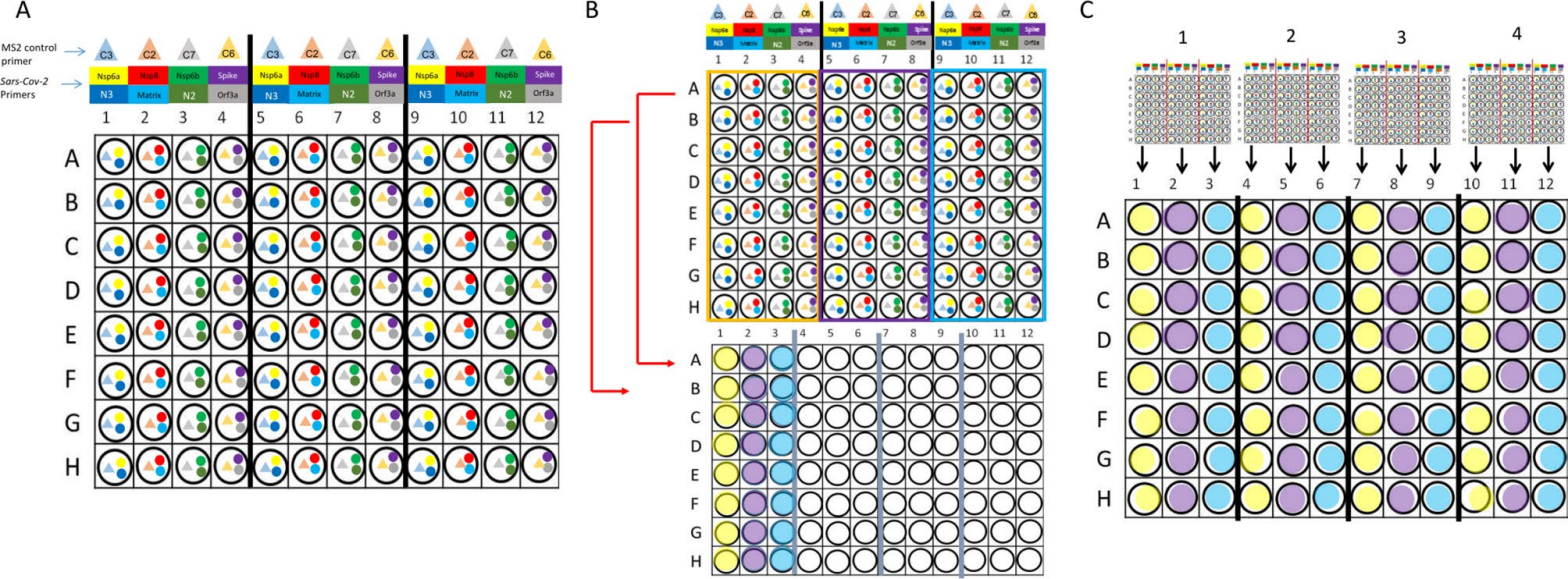
General scheme of how samples are processed in PCR and library plates (**A**) Each sample is individually amplified with a multiplex combination of primers for two SARS-CoV-2 targets and one MS2 control. (**B**) Every four columns of the PCR plate will be transformed into one column for the library plate. (**C**) Four complete PCR plaques (384 samples) generate one library plaque (96 individual sequencing libraries). Created with Microsoft Power Point.

Each patient’s set of individually amplified fragments for SARS-CoV-2 and MS2 genomes was combined in a pool using the Biomek FX Automation (Beckman Coulter). Please, refer to Fig. 5 for a scheme of how library pooling is performed. After pool combination, libraries were built using KAPA HiFi HotStart Enzyme (Roche) and Illumina Nextera XT v2 Index kit set A/B/C/D (Illumina, San Diego, CA, United States). Reaction was carried out with 5 µL cDNA, 25 µL KAPA HiFi Hot Start Enzyme, 5 µL Index i5, 5 µL Index i7, 10 µL nuclease-free water for a final reaction volume of 50 µL. The following cycles were used for the PCR reactions: 95 °C for 3 min, followed by 8 cycles of 95 °C for 30 s, 55 °C for 30 s and 72 °C for 5 min and followed by one cycle of 72 °C for 5 min.

### Library purification and normalization

Library purification was performed in the Biomek FX Automation (Beckman Coulter) using Agencourt AMPure XP Beads (Beckman Coulter) following standard protocols.

For normalization, all libraries were quantified by Qubit Fluorometer using Qubit™ dsDNA HS Assay Kit (Thermo Fisher Scientific, Waltham, MA) and size assessment was performed on Agilent 2200 TapeStation system for a dilution of 4 nM. Each 4 nM pool was combined into one pool and submitted to sequencing.

### Sequencing

Sample pools were diluted to 2 nM based on the Qiaseq Library Quant Assay Kit (Qiagen) measurements and the size information was performed on Agilent 2200 TapeStation system. For the pool denaturation, 10 µL of the 2 nM pool was added with 10 µL of 0.2 N NaOH, after 10uL de Tris–HCl pH7 200 mM was added to stop the denaturation process. 970 µL of Illumina’s HT1 buffer was added to the pool to dilute it to 20 pM and after 117 µL of 20 pM pool was added to 1.183 µL of Illumina’s HT1 buffer to dilute it to 1.8 pM. Diluted pool was spiked with 15% PhiX and sequenced using a Nextseq 500/550 Mid Reagent Cartridge v2 300 cycles (Illumina, San Diego, CA).

### Analyses

We developed an online platform to automate the entire process of sample registering, step check and display of results. This online platform was termed VarsVID, as it is a feature of the Varstation platform for genetic analyses (https://varstation.com/). General workflow of the entire process is defined as follows: (1) Wet-lab professional registers samples on VarsVID by uploading a CSV file containing sample ID and other optional data; (2) SampleSheet is generated by VarsVID and used for manual base calling and demultiplexer using Illumina’s tool blc2 fastq with barcode mismatches set to 0 and other parameters set to default; (3) FASTQ files are uploaded to a specific S3 bucket on AWS cloud; (4) An AWS lambda function is triggered to initiate a pre-defined EC2 instance, which will run the bioinformatics pipeline (explained in details bellow) and generate results; (5) Results are then processed by Python functions in the Django backend framework to generate user-friendly reports and display on VarsVID platform.

General steps of the bioinformatics pipeline that is executed in the EC2 instance is: (1) Quality Control, (2) Mapping to references (3) Assessing reads aligned to targets and (4) Demultiplexing to generate patient’s individual qualitative results. More specifically, Quality control is performed by cutadapt^16^ using filters to size (> 50 bp), average quality (Q_p_ > 20) and trimming options to remove NextSeq’s biased low-quality ends. Mapping to references was carried out using bwa mem tool^17^ (default parameters) with SARS-COV-2 isolate Wuhan-Hu-1 (NC_045512.2) and Phage MS2 (NC_001417.2) as pathogen reference and internal control spike-in, respectively. Reads aligned to the target were counted using Bedtools^18^ and qualitative results were generated based on a threshold of 10 reads or more and on the decision-table shown in Table 1. A quantitative value was calculated for each viral target based on abundance of reads. This metric was termed Fragments per Million (FPM) and is related to similar metrics used in the scientific community for NGS analyses (FPKM, RPKM, etc.). An additional normalization for amplicon length was not necessary, considering that all amplicons were designed to have the same size. In order to evaluate the relationship between CT and FPM values, a piecewise linear regression model was fit (Fig. 3). FPM values were log-transformed, since it is in principle a linear measure of viral load, while CT is a logarithmic measure. The analysis was carried out on PyMC3, an open-source Bayesian inference framework (Table 2). Further details about the statistical model and MCMC sampling are available as Supplemental Table S2. FPM is calculated as follows:

$$FPM = \frac{reads\_aligned\_target}{{passed\_QC\_reads}} \cdot 10^{6} .$$

**Table 2:** Decision table based on bioinformatics outputs to generate patient’s qualitative results.

Result	Control	Viral target 1	Viral target 2
Detected	+	+	+
Detected	−	+	+
Detected	+	+	−
Detected	+	−	+
Not detected	+	−	−
Repeat	−	−	−

A plus symbol indicates that the threshold of 10 reads was hitched for a specific SARS-CoV-2 or MS2 target, while a minus symbol indicates otherwise.

## Supplementary Information

Supplementary Information 1.

Supplementary Information 2.

Supplementary Information 3.

## Data Availability

Raw sequencing data for all samples tested in this work is publicly available through NCBI Sequence Read Archive (SRA) under the Bioproject PRJNA679460.
