# Supplementary material for: Mass molecular testing for COVID19 using NGS-based technology and a highly scalable workflow
Source: Sci Rep. 2021 Mar 29;11:7122. doi: 10.1038/s41598-021-86498-3 (PMC8007582; doi:10.1038/s41598-021-86498-3)
Supplement: Supplementary file 1 — Supplementary Information 1. [file 41598_2021_86498_MOESM1_ESM.pdf]

Mass molecular testing for COVID19 using NGS-based technology and a highly scalable workflow

Fernanda de Mello Malta[1*], Deyvid Amgarten[1*], Felipe Camilo Val[1], Murilo Castro Cervato[1], Bruna Mascaro Cordeiro de Azevedo[1], Marcela de Souza Basqueira[1], Camila Oliveira dos Santos Alves[1], Maria Soares Nobrega[1], Rodrigo de Souza Reis[1], Pedro Sebe[1], Michel Chieregato Gretschischkin[1], Diego Delgado Colombo de Oliveira[1], Carolina Naomi Izo Nakamura[1], Pedro Lui Nigro Chazanas[1], João Renato Rebello Pinho[1¥]

[1]Hospital Israelita Albert Einstein, São Paulo, Brazil
[*] Authors contributed equally to this work
[¥]Address for correspondence: João Renato Rebello Pinho, Laboratorio de Técnicas Especiais, Hospital Albert Einstein
email: joao.pinho@einstein.br

Table S1: Detailed description of turn-around times and lab analyst requested in each step of the workflow proposed in this work. We provide three different scenarios regarding the total number of samples in a 48h workflow.

Sample size	Step 1 - Sample screenig		Step 2 - Extraction plate preparation		Step 3 - Sample distribution		Step 4 - RNA extraction		Step 5 - cDNA + DNA amplification		Step 6 - Library preparation		Step 7 - Library purification		Step 8 - Library quantitation		Step 9 - Library normalization		Step 10 - Library Pooling		Step 11 – Pool quantitation (RT-qPCR)		Step 12 - Sequencing		Step 13 -Analysis		Total process time (hour)
	Lab Analyst	Time (hour)	Lab Analyst	Time (hour)	Lab Analyst	Time (hour)	Lab Analyst	Time (hour)	Lab Analyst	Time (hour)	Robot	Time (hour)	Robot	Time (hour)	Lab Analyst	Time (hour)	Lab Analyst	Time (hour)	Robot	Time (hour)	Lab Analyst	Time (hour)	Lab Analyst	Time (hour)	Lab Analyst	Time (hour)	
**1.500 Samples**	2	10.0	1	0.8	4	0.7	1	0.3	1	0.5	1	1.0	1	0.6	3	2.3	2	2.0	1	0.3	1	2.3	1	31.0	1	0.1	**51.9**
**3.000 Samples**	3	11.0	2	0.8	10	0.7	3	0.3	3	0.5	2	1.3	2	0.6	6	2.3	6	2.0	2	0.3	1	2.3	1	22.0	1	0.20	**44.3**
**6.000 Sample**	11	11.0	4	0.8	18	0.7	5	0.3	6	0.5	2	2.6	2	1.2	9	2.3	9	2.0	2	0.6	1	2.3	1	22.0	1	0.50	**46.8**
