# Supplementary material for: Mass molecular testing for COVID19 using NGS-based technology and a highly scalable workflow
Source: Sci Rep. 2021 Mar 29;11:7122. doi: 10.1038/s41598-021-86498-3 (PMC8007582; doi:10.1038/s41598-021-86498-3)
Supplement: Supplementary file 2 — Supplementary Information 2. [file 41598_2021_86498_MOESM2_ESM.docx]

**Mass molecular testing for COVID19 using NGS-based technology and a highly scalable workflow**

Fernanda de Mello Malta^1*^, Deyvid Amgarten^1*^, Felipe Camilo Val^1^, Murilo Castro Cervato^1^, Bruna Mascaro Cordeiro de Azevedo^1^, Marcela de Souza Basqueira^1^, Camila Oliveira dos Santos Alves^1^, Maria Soares Nobrega^1^, Rodrigo de Souza Reis^1^, Pedro Sebe^1^, Michel Chieregato Gretschischkin^1^, Diego Delgado Colombo de Oliveira^1^, Carolina Naomi Izo Nakamura^1^, Pedro Lui Nigro Chazanas^1^, João Renato Rebello Pinho^1¥^

^1^Hospital Israelita Albert Einstein, São Paulo, Brazil

^*^ Authors contributed equally to this work

^¥^Address for correspondence: João Renato Rebello Pinho, Laboratorio de Técnicas Especiais, Hospital Albert Einstein

email: joao.pinho@einstein.br

Table S2: *The statistical model used for correlating FPM and Ct is a piecewise linear model, Posterior distributions were estimated using Hamiltonian Monte Carlo (No U-Turn Sampler, with 10000 for tuning and 100000 samples for actual inference, divided between 10 Markov chains). All the model parameters had potential scale reduction factor (R-hat) under 1.001 and effective sample size above 7300, and there were no divergent transitions. The parameter labeled as threshold refers to the value of log FPM at which the regression line is allowed to change its slope and error standard deviation. Errors are assumed to be normally distributed*

| ***Parameter*** | ***Posterior mean*** | ***Credible interval (95% HDI)*** |
| --- | --- | --- |
| *Slope (high FPM)* | *-7.71* | *[-8.51, -6.93]* |
| *Slope (low FPM)* | *0.11* | *[-0.15, +0.37]* |
| *Intercept (high FPM)* | *60.1* | *[56.2, 64.2]* |
| *Intercept (low FPM)* | *29.2* | *[28.5, 29.9]* |
| *Error standard deviation (high FPM)* | *3.10* | *[2.65, 3.58]* |
| *Error standard deviation (low FPM)* | *1.27* | *[1.13, 1.41]* |
| *Threshold ** | *3.94* | *[3.83, 4.08]* |

***HDI: highest density interval***

Table S3. MS2 control primers with Illumina overhang.

| *Primers MS2* | Sequence |
| --- | --- |
| C2-1186F | 5’ -TCGTCGGCAGCGTCAGATGTGTATAAGAGACAGATCTCAGCCATGCATCGAGG- 3’ |
| C2-1465R | 5’ -GTCTCGTGGGCTCGGAGATGTGTATAAGAGACAGTTGTAAGCCTGTGAACGCGA- 3’ |
| C3-1653F | 5’ -TCGTCGGCAGCGTCAGATGTGTATAAGAGACAGAAGGCAATGCAAGGTCTCCT- 3’ |
| C3-1925R | 5’ -GTCTCGTGGGCTCGGAGATGTGTATAAGAGACAGCTTTGTGAGCAATTCGTCCCT- 3’ |
| C6-2048F | 5’ -TCGTCGGCAGCGTCAGATGTGTATAAGAGACAGCTTGTCATGGGATCCGGATGTTT- 3’ |
| C6-2309R | 5’ -GTCTCGTGGGCTCGGAGATGTGTATAAGAGACAGATATGACTCGTTATAGCGGACCG- 3’ |
| C7-2540F | 5’ -TCGTCGGCAGCGTCAGATGTGTATAAGAGACAGGTCTGCATCCGATTCCATCTCC- 3’ |
| C7-2812R | 5’ -GTCTCGTGGGCTCGGAGATGTGTATAAGAGACAGGGTGCAATCTCACTGGGACATAT- 3’ |

Table S4. Sars-Cov-2 target primers with Illumina overhang.

| Sars-Cov-2 *Primers* | Sequence |
| --- | --- |
| 11112_nsp6a_F | 5’ -TCGTCGGCAGCGTCAGATGTGTATAAGAGACAGTGGGTATTATTGCTATGTCTGCTT- 3’ |
| 11379_nsp6a_R | 5’ -GTCTCGTGGGCTCGGAGATGTGTATAAGAGACAGGCACCATCATCATACACAGTTCT- 3’ |
| 29178_N3_F | 5’ -TCGTCGGCAGCGTCAGATGTGTATAAGAGACAGACAAACATTGGCCGCAAATTG- 3’ |
| 29442_N3_R | 5’ -GTCTCGTGGGCTCGGAGATGTGTATAAGAGACAGTGTCTCTGCGGTAAGGCTTG- 3’ |
| 11447_nsp6b_F | 5’ -TCGTCGGCAGCGTCAGATGTGTATAAGAGACAGGATCAAGCCATTTCCATGTGGG- 3’ |
| 11686_nsp6b_R | 5’ -GTCTCGTGGGCTCGGAGATGTGTATAAGAGACAGAGTCAGTCTAAAGTAGCGGTTGA- 3’ |
| 28714_N2_F | 5’ -TCGTCGGCAGCGTCAGATGTGTATAAGAGACAGAGATCACATTGGCACCCGC- 3’ |
| 28973_N2_R | 5’ -GTCTCGTGGGCTCGGAGATGTGTATAAGAGACAGGCTGGTTCAATCTGTCAAGCA- 3’ |
| 22198_spike_F | 5’ -TCGTCGGCAGCGTCAGATGTGTATAAGAGACAGAGTGCGTGATCTCCCTCAGG- 3’ |
| 22461R_spike_R | 5’ -GTCTCGTGGGCTCGGAGATGTGTATAAGAGACAGAGAGGGTCAAGTGCACAGTC- 3’ |
| 25763_ORF3a_F | 5’ -TCGTCGGCAGCGTCAGATGTGTATAAGAGACAGTAATGAGGCTTTGGCTTTGCTG- 3’ |
| 26032_ORF3a_R | 5’ -GTCTCGTGGGCTCGGAGATGTGTATAAGAGACAGGCTGGTAATAGTCTGAAGTGAAGT- 3’ |
| 26758_matrix_F | 5’ -TCGTCGGCAGCGTCAGATGTGTATAAGAGACAGGGATCACCGGTGGAATTGCT- 3’ |
| 27021_matrix_R | 5’ -GTCTCGTGGGCTCGGAGATGTGTATAAGAGACAGCCTTGATGTCACAGCGTCCT- 3’ |
| 12230_nsp8_F | 5’ -TCGTCGGCAGCGTCAGATGTGTATAAGAGACAGTCTGAATTTGACCGTGATGCAG- 3’ |
| 12479_nsp8_R | 5’ -GTCTCGTGGGCTCGGAGATGTGTATAAGAGACAGCCATTAGTTTGGCTGCTGTTGT- 3’ |

Table S5. Primers combinations.

| **Sars-cov-2 primer 1** | **Sars-cov-2 primer 2** | **Control primer** |
| --- | --- | --- |
| Nsp6a | N3 | MS2-C3 |
| Nsp6b | N2 | MS2-C7 |
| Spike | ORF3a | MS2-C6 |
| Matrix | Nsp8 | MS2-C2 |
