# Supplementary material for: Mass molecular testing for COVID19 using NGS-based technology and a highly scalable workflow
Source: Sci Rep. 2021 Mar 29;11:7122. doi: 10.1038/s41598-021-86498-3 (PMC8007582; doi:10.1038/s41598-021-86498-3)
Supplement: Supplementary file 3 — Supplementary Information 3. [file 41598_2021_86498_MOESM3_ESM.pdf]

Mass molecular testing for COVID19 using NGS-based technology and a highly scalable workflow

Fernanda de Mello Malta[1*], Deyvid Amgarten[1*], Felipe Camilo Val[1], Murilo Castro Cervato[1], Bruna Mascaro Cordeiro de Azevedo[1], Marcela de Souza Basqueira[1], Camila Oliveira dos Santos Alves[1], Maria Soares Nobrega[1], Rodrigo de Souza Reis[1], Pedro Sebe[1], Michel Chieregato Gretschischkin[1], Diego Delgado Colombo de Oliveira[1], Carolina Naomi Izo Nakamura[1], Pedro Lui Nigro Chazanas[1], João Renato Rebello Pinho[1¥]

[1]Hospital Israelita Albert Einstein, São Paulo, Brazil
[*] Authors contributed equally to this work
[¥]Address for correspondence: João Renato Rebello Pinho, Laboratorio de Técnicas Especiais, Hospital Albert Einstein
email: joao.pinho@einstein.br

Folha de trabalho Coronavirus COVID-19

Data:	**12/11/2020 09:46**	Multi, Index:	**Multi_1, A**
Analista:	____________	Placa Extração/Amplificação:	**20201112-1**

	C3-nsp6a-N3 1	C2-nsp8-matrix 2	C7-nsp6b-N2 3	C6-spike-ORF3a 4	C3-nsp6a-N3 5	C2-nsp8-matrix 6	C7-nsp6b-N2 7	C6-spike-ORF3a 8	C3-nsp6a-N3 9	C2-nsp8-matrix 10	C7-nsp6b-N2 11	C6-spike-ORF3a 12
**A**	**ID_001** Patient E 0	**ID_002** Patient E 0	**ID_003** Patient E 0	**ID_004** Patient E 0	**ID_005** Patient E 0	**ID_006** Patient E 0	**ID_007** Patient E 0	**ID_008** Patient E 0	**ID_009** Patient E 0	**ID_010** Patient E 0	**ID_011** Patient E 0	**ID_012** Patient E 0
**B**	**ID_013** Patient E 0	**ID_014** Patient E 0	**ID_015** Patient E 0	**ID_016** Patient E 0	**ID_017** Patient E 0	**ID_018** Patient E 0	**ID_019** Patient E 0	**ID_020** Patient E 0	**ID_021** Patient E 0	**ID_022** Patient E 0	**ID_023** Patient E 0	**ID_024** Patient E 0
**C**	**ID_025** Patient E 0	**ID_026** Patient E 0	**ID_027** Patient E 0	**ID_028** Patient E 0	**CN**	**ID_029** Patient E 0	**ID_030** Patient E 0	**ID_031** Patient E 0	**ID_032** Patient E 0	**ID_033** Patient E 0	**ID_034** Patient E 0	**ID_035** Patient E 0
**D**	**ID_036** Patient E 0	**ID_037** Patient E 0	**ID_038** Patient E 0	**ID_039** Patient E 0	**ID_040** Patient E 0	**ID_041** Patient E 0	**ID_042** Patient E 0	**ID_043** Patient E 0	**ID_044** Patient E 0	**ID_045** Patient E 0	**ID_046** Patient E 0	**ID_047** Patient E 0
**E**	**ID_048** Patient E 0	**ID_049** Patient E 0	**ID_050** Patient E 0	**ID_051** Patient E 0	**ID_052** Patient E 0	**ID_053** Patient E 0	**ID_054** Patient E 0	**ID_055** Patient E 0	**ID_056** Patient E 0	**ID_057** Patient E 0	**ID_058** Patient E 0	**ID_059** Patient E 0
**F**	**ID_060** Patient E 0	**ID_061** Patient E 0	**ID_062** Patient E 0	**ID_063** Patient E 0	**ID_064** Patient E 0	**ID_065** Patient E 0	**ID_066** Patient E 0	**ID_067** Patient E 0	**ID_068** Patient E 0	**ID_069** Patient E 0	**ID_070** Patient E 0	**ID_071** Patient E 0
**G**	**ID_072** Patient E 0	**ID_073** Patient E 0	**ID_074** Patient E 0	**ID_075** Patient E 0	**ID_076** Patient E 0	**ID_077** Patient E 0	**ID_078** Patient E 0	**ID_079** Patient E 0	**ID_080** Patient E 0	**ID_081** Patient E 0	**ID_082** Patient E 0	**ID_083** Patient E 0
**H**	**ID_084** Patient E 0	**ID_085** Patient E 0	**ID_086** Patient E 0	**ID_087** Patient E 0	**ID_088** Patient E 0	**ID_089** Patient E 0	**ID_090** Patient E 0	**ID_091** Patient E 0	**ID_092** Patient E 0	**ID_093** Patient E 0	**ID_094** Patient E 0	**ID_095** Patient E 0

Data: **12/11/2020 09:46** Multi, Index: **Multi_1, A**

Analista: ____________________ Placa Extração/Amplificação: **20201112-2**

	C3-nsp6a-N3 1	C2-nsp8-matrix 2	C7-nsp6b-N2 3	C6-spike-ORF3a 4	C3-nsp6a-N3 5	C2-nsp8-matrix 6	C7-nsp6b-N2 7	C6-spike-ORF3a 8	C3-nsp6a-N3 9	C2-nsp8-matrix 10	C7-nsp6b-N2 11	C6-spike-ORF3a 12
A	ID_096 Patient E 0	ID_097 Patient E 0	ID_098 Patient E 0	ID_099 Patient E 0	ID_100 Patient E 1	ID_101 Patient E 1	ID_102 Patient E 1	ID_103 Patient E 1	ID_104 Patient E 1	ID_105 Patient E 1	ID_106 Patient E 1	ID_107 Patient E 1
B	ID_108 Patient E 1	ID_109 Patient E 1	ID_110 Patient E 1	ID_111 Patient E 1	ID_112 Patient E 1	ID_113 Patient E 1	ID_114 Patient E 1	ID_115 Patient E 1	ID_116 Patient E 1	ID_117 Patient E 1	ID_118 Patient E 1	ID_119 Patient E 1
C	ID_120 Patient E 1	ID_121 Patient E 1	ID_122 Patient E 1	ID_123 Patient E 1	ID_124 Patient E 1	ID_125 Patient E 1	ID_126 Patient E 1	ID_127 Patient E 1	ID_128 Patient E 1	ID_129 Patient E 1	ID_130 Patient E 1	ID_131 Patient E 1
D	ID_132 Patient E 1	ID_133 Patient E 1	ID_134 Patient E 1	ID_135 Patient E 1	ID_136 Patient E 1	ID_137 Patient E 1	ID_138 Patient E 1	ID_139 Patient E 1	ID_140 Patient E 1	ID_141 Patient E 1	ID_142 Patient E 1	ID_143 Patient E 1
E	ID_144 Patient E 1	ID_145 Patient E 1	ID_146 Patient E 1	ID_147 Patient E 1	ID_148 Patient E 1	ID_149 Patient E 1	ID_150 Patient E 1	ID_151 Patient E 1	ID_152 Patient E 1	ID_153 Patient E 1	ID_154 Patient E 1	ID_155 Patient E 1
F	ID_156 Patient E 1	CN	ID_157 Patient E 1	ID_158 Patient E 1	ID_159 Patient E 1	ID_160 Patient E 1	ID_161 Patient E 1	ID_162 Patient E 1	ID_163 Patient E 1	ID_164 Patient E 1	ID_165 Patient E 1	ID_166 Patient E 1
G	ID_167 Patient E 1	ID_168 Patient E 1	ID_169 Patient E 1	ID_170 Patient E 1	ID_171 Patient E 1	ID_172 Patient E 1	ID_173 Patient E 1	ID_174 Patient E 1	ID_175 Patient E 1	ID_176 Patient E 1	ID_177 Patient E 1	ID_178 Patient E 1
H	ID_179 Patient E 1	ID_180 Patient E 1	ID_181 Patient E 1	ID_182 Patient E 1	ID_183 Patient E 1	ID_184 Patient E 1	ID_185 Patient E 1	ID_186 Patient E 1	ID_187 Patient E 1	ID_188 Patient E 1	ID_189 Patient E 1	ID_190 Patient E 1

Data:	**12/11/2020 09:46**	Multi, Index:	**Multi_1, A**
Analista:	______________________	Placa Extração/Amplificação:	**20201112-3**

	**C3-nsp6a-N3 1**	**C2-nsp8-matrix 2**	**C7-nsp6b-N2 3**	**C6-spike-ORF3a 4**	**C3-nsp6a-N3 5**	**C2-nsp8-matrix 6**	**C7-nsp6b-N2 7**	**C6-spike-ORF3a 8**	**C3-nsp6a-N3 9**	**C2-nsp8-matrix 10**	**C7-nsp6b-N2 11**	**C6-spike-ORF3a 12**
**A**	**ID_191** Patient E 1	**ID_192** Patient E 1	**ID_193** Patient E 1	**ID_194** Patient E 1	**ID_195** Patient E 1	**ID_196** Patient E 1	**ID_197** Patient E 1	**ID_198** Patient E 1	**ID_199** Patient E 1	**ID_200** Patient E 2	**ID_201** Patient E 2	**ID_202** Patient E 2
**B**	**ID_203** Patient E 2	**ID_204** Patient E 2	**ID_205** Patient E 2	**ID_206** Patient E 2	**ID_207** Patient E 2	**ID_208** Patient E 2	**ID_209** Patient E 2	**ID_210** Patient E 2	**ID_211** Patient E 2	**ID_212** Patient E 2	**ID_213** Patient E 2	**ID_214** Patient E 2
**C**	**ID_215** Patient E 2	**ID_216** Patient E 2	**ID_217** Patient E 2	**ID_218** Patient E 2	**ID_219** Patient E 2	**ID_220** Patient E 2	**ID_221** Patient E 2	**ID_222** Patient E 2	**ID_223** Patient E 2	**ID_224** Patient E 2	**ID_225** Patient E 2	**ID_226** Patient E 2
**D**	**ID_227** Patient E 2	**ID_228** Patient E 2	**ID_229** Patient E 2	**ID_230** Patient E 2	**ID_231** Patient E 2	**ID_232** Patient E 2	**ID_233** Patient E 2	**ID_234** Patient E 2	**ID_235** Patient E 2	**ID_236** Patient E 2	**ID_237** Patient E 2	**ID_238** Patient E 2
**E**	**ID_239** Patient E 2	**ID_240** Patient E 2	**ID_241** Patient E 2	**ID_242** Patient E 2	**ID_243** Patient E 2	**ID_244** Patient E 2	**ID_245** Patient E 2	**ID_246** Patient E 2	**ID_247** Patient E 2	**ID_248** Patient E 2	**ID_249** Patient E 2	**ID_250** Patient E 2
**F**	**ID_251** Patient E 2	**ID_252** Patient E 2	**ID_253** Patient E 2	**ID_254** Patient E 2	**ID_255** Patient E 2	**ID_256** Patient E 2	**ID_257** Patient E 2	**ID_258** Patient E 2	**ID_259** Patient E 2	**ID_260** Patient E 2	**ID_261** Patient E 2	**ID_262** Patient E 2
**G**	**ID_263** Patient E 2	**ID_264** Patient E 2	**ID_265** Patient E 2	**ID_266** Patient E 2	**ID_267** Patient E 2	**ID_268** Patient E 2	**ID_269** Patient E 2	**ID_270** Patient E 2	**ID_271** Patient E 2	**ID_272** Patient E 2	**ID_273** Patient E 2	**ID_274** Patient E 2
**H**	**ID_275** Patient E 2	**ID_276** Patient E 2	**ID_277** Patient E 2	**ID_278** Patient E 2	**ID_279** Patient E 2	**ID_280** Patient E 2	**ID_281** Patient E 2	**ID_282** Patient E 2	**ID_283** Patient E 2	**ID_284** Patient E 2	**CN**	**ID_285** Patient E 2

Data:	**12/11/2020 09:46**	Multi, Index:	**Multi_1, A**
Analista:	______________	Placa Extração/Amplificação:	**20201112-4**

	C3-nsp6a-N3 1	C2-nsp8-matrix 2	C7-nsp6b-N2 3	C6-spike-ORF3a 4	C3-nsp6a-N3 5	C2-nsp8-matrix 6	C7-nsp6b-N2 7	C6-spike-ORF3a 8	C3-nsp6a-N3 9	C2-nsp8-matrix 10	C7-nsp6b-N2 11	C6-spike-ORF3a 12
**A**	**ID_286** Patient E 2	**ID_287** Patient E 2	**ID_288** Patient E 2	**ID_289** Patient E 2	**ID_290** Patient E 2	**ID_291** Patient E 2	**ID_292** Patient E 2	**CN**	**ID_293** Patient E 2	**ID_294** Patient E 2	**ID_295** Patient E 2	**ID_296** Patient E 2
**B**	**ID_297** Patient E 2	**ID_298** Patient E 2	**ID_299** Patient E 2	**ID_300** Patient E 3	**ID_301** Patient E 3	**ID_302** Patient E 3	**ID_303** Patient E 3	**ID_304** Patient E 3	**ID_305** Patient E 3	**ID_306** Patient E 3	**ID_307** Patient E 3	**ID_308** Patient E 3
**C**	**ID_309** Patient E 3	**ID_310** Patient E 3	**ID_311** Patient E 3	**ID_312** Patient E 3	**ID_313** Patient E 3	**ID_314** Patient E 3	**ID_315** Patient E 3	**ID_316** Patient E 3	**ID_317** Patient E 3	**ID_318** Patient E 3	**ID_319** Patient E 3	**ID_320** Patient E 3
**D**	**ID_321** Patient E 3	**ID_322** Patient E 3	**ID_323** Patient E 3	**ID_324** Patient E 3	**ID_325** Patient E 3	**ID_326** Patient E 3	**ID_327** Patient E 3	**ID_328** Patient E 3	**ID_329** Patient E 3	**ID_330** Patient E 3	**ID_331** Patient E 3	**ID_332** Patient E 3
**E**	**ID_333** Patient E 3	**ID_334** Patient E 3	**ID_335** Patient E 3	**ID_336** Patient E 3	**ID_337** Patient E 3	**ID_338** Patient E 3	**ID_339** Patient E 3	**ID_340** Patient E 3	**ID_341** Patient E 3	**ID_342** Patient E 3	**ID_343** Patient E 3	**ID_344** Patient E 3
**F**	**ID_345** Patient E 3	**ID_346** Patient E 3	**ID_347** Patient E 3	**ID_348** Patient E 3	**ID_349** Patient E 3	**ID_350** Patient E 3	**ID_351** Patient E 3	**ID_352** Patient E 3	**ID_353** Patient E 3	**ID_354** Patient E 3	**ID_355** Patient E 3	**ID_356** Patient E 3
**G**	**ID_357** Patient E 3	**ID_358** Patient E 3	**ID_359** Patient E 3	**ID_360** Patient E 3	**ID_361** Patient E 3	**ID_362** Patient E 3	**ID_363** Patient E 3	**ID_364** Patient E 3	**ID_365** Patient E 3	**ID_366** Patient E 3	**ID_367** Patient E 3	**ID_368** Patient E 3
**H**	**ID_369** Patient E 3	**ID_370** Patient E 3	**ID_371** Patient E 3	**ID_372** Patient E 3	**ID_373** Patient E 3	**ID_374** Patient E 3	**ID_375** Patient E 3	**ID_376** Patient E 3	**ID_377** Patient E 3	**ID_378** Patient E 3	**ID_379** Patient E 3	**ID_380** Patient E 3

Folha de trabalho Coronavirus COVID-19

Data:	**12/11/2020 09:46**	Multi, Index:	**Multi_1, A**
Analista:	______________________	Placa Extração/Amplificação:	**20201112-1, 20201112-2, 20201112-3, 20201112-4**

	**Placa Multi - Combinação Indices A 1**	**Placa Multi - Combinação Indices A 2**	**Placa Multi - Combinação Indices A 3**	**Placa Multi - Combinação Indices A 4**	**Placa Multi - Combinação Indices A 5**	**Placa Multi - Combinação Indices A 6**	**Placa Multi - Combinação Indices A 7**	**Placa Multi - Combinação Indices A 8**	**Placa Multi - Combinação Indices A 9**	**Placa Multi - Combinação Indices A 10**	**Placa Multi - Combinação Indices A 11**	**Placa Multi - Combinação Indices A 12**
**A**	ID_001<br>ID_002<br>ID_003<br>ID_00	ID_005<br>ID_006<br>ID_007<br>ID_00	ID_009<br>ID_010<br>ID_011<br>ID_01	ID_096<br>ID_097<br>ID_098<br>ID_09	ID_100<br>ID_101<br>ID_102<br>ID_10	ID_104<br>ID_105<br>ID_106<br>ID_10	ID_191<br>ID_192<br>ID_193<br>ID_19	ID_195<br>ID_196<br>ID_197<br>ID_19	ID_199<br>ID_200<br>ID_201<br>ID_20	ID_286<br>ID_287<br>ID_288<br>ID_28	ID_290<br>ID_291<br>ID_292<br>-	ID_293<br>ID_294<br>ID_295<br>ID_29
**B**	ID_013<br>ID_014<br>ID_015<br>ID_01	ID_017<br>ID_018<br>ID_019<br>ID_02	ID_021<br>ID_022<br>ID_023<br>ID_02	ID_108<br>ID_109<br>ID_110<br>ID_11	ID_112<br>ID_113<br>ID_114<br>ID_11	ID_116<br>ID_117<br>ID_118<br>ID_11	ID_203<br>ID_204<br>ID_205<br>ID_20	ID_207<br>ID_208<br>ID_209<br>ID_21	ID_211<br>ID_212<br>ID_213<br>ID_21	ID_297<br>ID_298<br>ID_299<br>ID_30	ID_301<br>ID_302<br>ID_303<br>ID_30	ID_305<br>ID_306<br>ID_307<br>ID_30
**C**	ID_025<br>ID_026<br>ID_027<br>ID_02	- -<br>ID_029<br>ID_030<br>ID_03	ID_032<br>ID_033<br>ID_034<br>ID_03	ID_120<br>ID_121<br>ID_122<br>ID_12	ID_124<br>ID_125<br>ID_126<br>ID_12	ID_128<br>ID_129<br>ID_130<br>ID_13	ID_215<br>ID_216<br>ID_217<br>ID_21	ID_219<br>ID_220<br>ID_221<br>ID_22	ID_223<br>ID_224<br>ID_225<br>ID_22	ID_309<br>ID_310<br>ID_311<br>ID_31	ID_313<br>ID_314<br>ID_315<br>ID_31	ID_317<br>ID_318<br>ID_319<br>ID_32
**D**	ID_036<br>ID_037<br>ID_038<br>ID_03	ID_040<br>ID_041<br>ID_042<br>ID_04	ID_044<br>ID_045<br>ID_046<br>ID_04	ID_132<br>ID_133<br>ID_134<br>ID_13	ID_136<br>ID_137<br>ID_138<br>ID_13	ID_140<br>ID_141<br>ID_142<br>ID_14	ID_227<br>ID_228<br>ID_229<br>ID_23	ID_231<br>ID_232<br>ID_233<br>ID_23	ID_235<br>ID_236<br>ID_237<br>ID_23	ID_321<br>ID_322<br>ID_323<br>ID_32	ID_325<br>ID_326<br>ID_327<br>ID_32	ID_329<br>ID_330<br>ID_331<br>ID_33
**E**	ID_048<br>ID_049<br>ID_050<br>ID_05	ID_052<br>ID_053<br>ID_054<br>ID_05	ID_056<br>ID_057<br>ID_058<br>ID_05	ID_144<br>ID_145<br>ID_146<br>ID_14	ID_148<br>ID_149<br>ID_150<br>ID_15	ID_152<br>ID_153<br>ID_154<br>ID_15	ID_239<br>ID_240<br>ID_241<br>ID_24	ID_243<br>ID_244<br>ID_245<br>ID_24	ID_247<br>ID_248<br>ID_249<br>ID_25	ID_333<br>ID_334<br>ID_335<br>ID_33	ID_337<br>ID_338<br>ID_339<br>ID_34	ID_341<br>ID_342<br>ID_343<br>ID_34
**F**	ID_060<br>ID_061<br>ID_062<br>ID_06	ID_064<br>ID_065<br>ID_066<br>ID_06	ID_068<br>ID_069<br>ID_070<br>ID_07	ID_156<br>- -<br>ID_157<br>ID_15	ID_159<br>ID_160<br>ID_161<br>ID_16	ID_163<br>ID_164<br>ID_165<br>ID_16	ID_251<br>ID_252<br>ID_253<br>ID_25	ID_255<br>ID_256<br>ID_257<br>ID_25	ID_259<br>ID_260<br>ID_261<br>ID_26	ID_345<br>ID_346<br>ID_347<br>ID_34	ID_349<br>ID_350<br>ID_351<br>ID_35	ID_353<br>ID_354<br>ID_355<br>ID_35
**G**	ID_072<br>ID_073<br>ID_074<br>ID_07	ID_076<br>ID_077<br>ID_078<br>ID_07	ID_080<br>ID_081<br>ID_082<br>ID_08	ID_167<br>ID_168<br>ID_169<br>ID_17	ID_171<br>ID_172<br>ID_173<br>ID_17	ID_175<br>ID_176<br>ID_177<br>ID_17	ID_263<br>ID_264<br>ID_265<br>ID_26	ID_267<br>ID_268<br>ID_269<br>ID_27	ID_271<br>ID_272<br>ID_273<br>ID_27	ID_357<br>ID_358<br>ID_359<br>ID_36	ID_361<br>ID_362<br>ID_363<br>ID_36	ID_365<br>ID_366<br>ID_367<br>ID_36
**H**	ID_084<br>ID_085<br>ID_086<br>ID_08	ID_088<br>ID_089<br>ID_090<br>ID_09	ID_092<br>ID_093<br>ID_094<br>ID_09	ID_179<br>ID_180<br>ID_181<br>ID_18	ID_183<br>ID_184<br>ID_185<br>ID_18	ID_187<br>ID_188<br>ID_189<br>ID_19	ID_275<br>ID_276<br>ID_277<br>ID_27	ID_279<br>ID_280<br>ID_281<br>ID_28	ID_283<br>ID_284<br>- -<br>ID_28	ID_369<br>ID_370<br>ID_371<br>ID_37	ID_373<br>ID_374<br>ID_375<br>ID_37	ID_377<br>ID_378<br>ID_379<br>ID_38
